# Effects of 1-year exercise in patients with atrial fibrillation: study protocol for the Norwegian Exercise in Atrial Fibrillation (NEXAF) randomised controlled trial

**DOI:** 10.1136/openhrt-2024-003077

**Published:** 2025-03-22

**Authors:** Bjarne M Nes, Jon Magne Letnes, Kristin Espolin Johnson, Andreas Berg Sellevold, Rune Byrkjeland, Fedelix Phetogo Brown, Turid Follestad, Håvard Dalen, Ulrik Wisløff, Maja-Lisa Løchen, Arnljot Tveit, Bente Morseth, Marius Myrstad, Jan Pål Loennechen, The NEXAF consortium, Bente Morseth

**Affiliations:** 1 Department of Circulation and Medical Imaging, Norwegian University of Science and Technology, Trondheim, Norway; 2 Clinic of Cardiology, St Olavs Hospital University Hospital, Trondheim, Norway; 3 Department of Medical Research Bærum Hospital, Vestre Viken Hospital Trust, Gjettum, Norway; 4 Department of Internal Medicine, Vestre Viken Hospital Trust, Gjettum, Norway; 5 Department of Cardiology, University Hospital of North Norway, Tromso, Norway; 6 Department of Public Health and Nursing, Norwegian University of Science and Technology, Trondheim, Norway; 7 Department of Medicine, Levanger Hospital, Nord-Trøndelag Hospital Trust, Levanger, Norway; 8 Department of Clinical Medicine, UiT The Arctic University of Norway, Tromso, Norway; 9 Faculty of Medicine, Institute of Clinical Medicine, University of Oslo, Oslo, Norway; 10 School of Sport Sciences, UiT The Arctic University of Norway, Tromsø, Norway

**Keywords:** Atrial Fibrillation, Cardiac Rehabilitation, Outcome Assessment, Health Care

## Abstract

**Introduction:**

Atrial fibrillation is the most prevalent sustained arrhythmia worldwide and is expected to increase substantially within the coming years. Although lifestyle changes and risk factor modification are now acknowledged as central components of atrial fibrillation management, the effects of exercise on disease-specific outcomes are still not extensively documented due to few high-quality randomised trials. The primary objective of the Norwegian Exercise in Atrial Fibrillation Trial (NEXAF) is to assess the effects of exercise over 12 months on key clinical and patient-reported outcomes in previously inactive patients with atrial fibrillation.

**Methods and analysis:**

NEXAF is a multicentre, two-arm, randomised controlled trial inviting patients 18–80 years with a confirmed diagnosis of paroxysmal or persistent atrial fibrillation. Eligible patients are randomised 1:1 to either a combined supervised and eHealth-based exercise intervention or usual care for 12 months. The primary outcomes are total time in atrial fibrillation measured by insertable cardiac monitors, and disease-specific quality of life measured by the Atrial Fibrillation Effect on Quality-of-Life questionnaire.

**Ethics and dissemination:**

Ethical approval was obtained from the Regional Ethics Committee in Mid-Norway in April 2021 (ID 213848).

**Trial registration number:**

NCT05164718.

**WHAT IS ALREADY KNOWN ON THIS TOPIC**

- Lifestyle recommendations are acknowledged as a key component of optimal atrial fibrillation (AF) management, with tailored exercise suggested as a key target.
- AF patients are less physically active than those without AF, and exercise-based cardiac rehabilitation is not routinely available.

**WHAT THIS STUDY ADDS**

- This is the largest clinical trial to date that examines the effects of long-term exercise on clinical and patient-reported outcomes in patients with AF.
- Use of implantable loop recorders allows for precise quantification of AF burden contrary to binary evaluation of AF recurrence.

**HOW THIS STUDY MIGHT AFFECT RESEARCH, PRACTICE OR POLICY**

- This trial will provide robust documentation of the effects of exercise in AF management.
- The long-term, hybrid (centre and home-based) intervention is pragmatic and feasible, and thus readily available for clinical implementation.

## Introduction

 Atrial fibrillation (AF) is the most common clinically relevant arrhythmia, estimated to affect more than 50 million people worldwide.^1 2^ Recent projections estimate that the global AF prevalence may increase by more than 60% within 2050.^1^ The increase is attributed to ageing populations, as well as an increase in common AF risk factors such as obesity and concomitant cardiovascular diseases (CVDs). Further, the fast-growing use of user-led wearable technology for arrhythmia detection, and the potential for mass screening via new medical devices, may lead to earlier detection and further increase the number of AF diagnoses. In addition, these devices allow for long-term remote monitoring and more accurate quantification of total AF burden, which may be used to refine and individualise future treatment and management of patients.^3^ For example, the risk of stroke and heart failure is associated with AF burden and may together with comorbidity burden and lifestyle factors impact decisions on treatment strategy.^3^

Moreover, most AF patients are symptomatic with palpitations, exercise intolerance and dyspnoea as common symptoms and report reduced quality of life (QoL). Evaluating symptom burden and QoL through patient-reported outcome measures is, therefore, key to guide and assess patient management.^4^ Being diagnosed with AF is associated with frequent hospitalisations and increased risk of stroke, heart failure and other CVDs.^4^ In this context, effective and targeted treatment and secondary preventive measures are necessary to ease the burden for AF patients and to reduce future healthcare costs.

Exercise is recommended in both primary and secondary CVD prevention and in cardiac rehabilitation and provides numerous well-documented health benefits with generally few side effects.^5 6^ Its role in AF prevention and management is, however, ambiguous. Sustained high-volume endurance exercise is associated with increased risk of AF, while moderate physical activity (PA) at the level of the general recommendations seems protective.^7^ The evidence is more limited among patients with established AF. Observational data suggest that AF patients who report exercise according to the general recommendations have reduced risk of premature mortality and clinical events such as stroke, heart failure and myocardial infarction.^8 9^ The extent to which the risk reductions observed with PA are related to the AF pattern or burden per se is however unknown.

Among clinical trials, several small-scale studies indicate that exercise may have beneficial effects on QoL, symptom burden and exercise capacity.^10^,^12^ A 12-week randomised controlled trial that compared high-intensity interval exercise to standard treatment in patients with paroxysmal AF (n=51), reported significantly reduced time-in-AF measured by insertable cardiac monitors (ICMs) among patients in the exercise group.^13^ The study was, however, limited by a small sample size and relatively short follow-up. Recently, the findings were supported by the larger ACTIVE-AF study (n=120) that reported reduced symptom severity and lower rate of AF recurrence by Holter monitoring after 6 months of combined supervised and home-based exercise, compared with control, in patients with symptomatic paroxysmal or persistent AF.^14^

Following the emerging evidence of exercise benefits, the recent European^4^ and US^15^ guidelines for management of AF now state moderate-to-vigorous exercise in non-permanent AF patients as a class I recommendation. Still, clinical exercise trials in AF patients are scarce and with relatively few total included patients. Hence, the latest Cochrane review concluded that high-quality randomised controlled trials are needed and should prioritise patient-relevant measures such as AF burden, symptom severity and AF-specific QoL.^16^ Exercise strategies and approaches that are proven feasible and effective for AF patients in the long term, and thus could be readily implemented, are also lacking. Therefore, exercise prescription or rehabilitation programmes are not yet routinely available for AF patients. These are major concerns as most AF patients are less active than recommended and have a high burden of cardiovascular risk factors and comorbidities,^17^ suggesting that exercise would be beneficial and should be a central part of rehabilitation and secondary prevention.

The overall aim of the Norwegian Exercise in Atrial Fibrillation (NEXAF) trial is, therefore, to provide robust documentation of the effects of long-term exercise on patient-relevant outcomes, for future evidence-based exercise recommendations and prescription for AF patients. NEXAF will test a pragmatic and feasible long-term intervention with use of remote and continuous monitoring of both exercise levels and arrythmia burden through commercially available devices. Hence, our design may create both robust evidence and opportunities for broad application and clinical implementation.

### Study objectives and hypotheses

The primary objective of NEXAF is to determine whether a structured, combined supervised and eHealth-based, exercise intervention in formerly inactive patients is superior to usual care alone in reducing AF burden and improving disease-specific QoL, measured by time in AF by an ICM, and the overall sum score of the Atrial Fibrillation Effect on Quality of Life (AFEQT) questionnaire.

As secondary objectives NEXAF will assess the effects of exercise on frequency and duration of AF episodes, symptoms burden, general health, peak oxygen uptake (VO2peak), cardiac structure and function, and cardiovascular risk factors.

## Methods and analysis

### Study design

**Figure 1:**
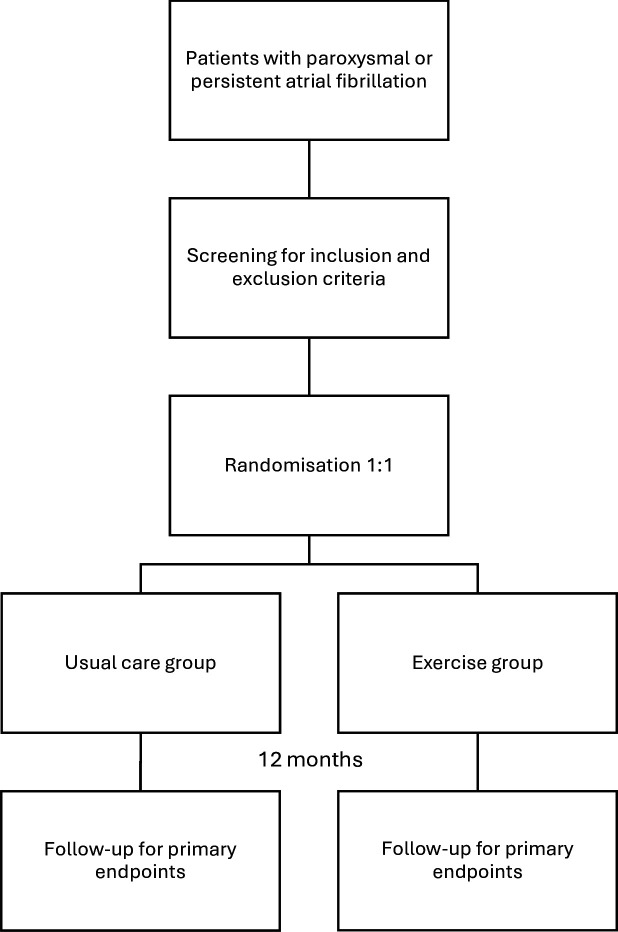
Brief overview of the NEXAF trial design. NEXAF, Norwegian Exercise in Atrial Fibrillation Trial.

NEXAF is a prospective, two-arm, randomised, open-label multicentre trial including patients with paroxysmal or persistent AF. Eligible patients are randomised to receive either usual care only, or a combined supervised and eHealth-based exercise intervention for 12 months (figure 1). Study recruitment started in February 2022 and randomisation was concluded by October 2024. The study is initiated and coordinated by the Norwegian University of Science and Technology (NTNU) and Clinic of Cardiology at St. Olav’s hospital, Trondheim, Norway. Participating centres are Bærum Hospital, Vestre Viken Hospital Trust and the University Hospital of North Norway in collaboration with UiT The Arctic University of Norway, Tromsø.

### Study population, screening and randomisation

The target population for the NEXAF trial is previously inactive (report less the minimum recommended volume) patients with a confirmed diagnose of paroxysmal or persistent AF who are managed with a rhythm control strategy. Further eligibility criteria are mainly set to ensure either safety or practicality of a long-term exercise intervention. Eligible patients are identified through International Classification of Diseases version 10 codes (I48) at the hospitals’ inpatient and outpatient registries and screened through hospital records and by interview by a study physician. The screening criteria are outlined in table 1. All included patients must sign an informed consent form prior to enrolment and are then provided an ICM (Confirm RX, Abbott) allowing continuous AF monitoring. At baseline visits, eligible patients must be in sinus rhythm for examinations to be performed. If ongoing AF a new appointment will be made as soon as the patient reports sinus rhythm or within maximally 6 weeks. Patients still in AF after 6 weeks will not be included, but a new evaluation can be performed if sinus rhythm is obtained within 3 months. After completing baseline examinations, patients are randomised with a 1:1 allocation ratio, by a computer-generated schedule stratified by recruiting centre and paroxysmal versus persistent AF, to an exercise intervention or usual care (control).

**Table 1:** Inclusion and exclusion criteria for the NEXAF trial

Inclusion criteria	Exclusion criteria
Age ≥18 years and <80 years	Permanent or persistent AF with at least one continuous episode lasting ≥3 months the last year
Live within a reasonable distance to the nearest study centre (appr. 1 hour)	AF as a complication of acute coronary syndromes, cardiothoracic surgery or infections
Diagnosed with paroxysmal or persistent AF (ICD10-I48) in hospital registries with an ECG available for review confirming AF	Planned ablation procedure next 12 months or ablated last 6 months without known recurrence
In sinus rhythm at baseline examinations	Unstable coronary heart disease or hospitalised for coronary heart disease last 3 months
Report <150 min per week of moderate and/or <75 min vigorous exercise the last 3 months	Decompensated heart failure or hospitalised for heart failure last 3 months
Own and use a smartphone	Left ventricular ejection fraction <40%
	At least moderate to severe mitral or aortic pathology, mechanical valves or aortic aneurysms
	Moderate to severe chronic obstructive pulmonary disease (GOLD group C+D)
	Ongoing severe cancer or active cancer treatment
	Pacemaker or ICD
	Pregnancy
	Alcohol or drug abuse
	Cognitive or serious psychiatric disease that may impede protocol compliance
	Physical impairments or diseases hindering exercise or making exercise or implantation contraindicated
	Resident of nursing home or other institution
	Participation in conflicting research studies (ie, lifestyle interventions)

AFatrial fibrillationICDimplantable cardiac deviceICD-10International Classification of Diseases version 10NEXAFNorwegian Exercise in Atrial Fibrillation Trial

### Study intervention

#### Exercise group

Patients in the exercise group are instructed to attain 150–300 min of moderate-intensity activity (55%–75% of maximum heart rate in sinus rhythm or rated perceived exertion at 12–13 on the Borg Scale) or 75–150 min of vigorous-intensity activity (75%–95% of maximum heart rate or above 14 on the Borg Scale) per week, or an equivalent combination of both, according to current general PA recommendations. They are also encouraged to obtain at least 20–30 min of the weekly total with vigorous intensity. Strength training is optional, with no instruction given, and should be performed in addition to aerobic exercise.

During the first month, participants are introduced to the exercise programme through 2 weekly practice sessions, individually or in small groups of 2–4 participants, under supervision by a physiotherapist or exercise physiologist. Participants are given a smart watch (Garmin Venu SQ, Garmin) for monitoring of activity and heart rate during exercise, that are introduced and used during sessions. The initial supervised sessions focus on aerobic interval training (4 times 4 min bouts at 85%–95% of maximal heart rate) on treadmill, bicycle or outdoors. After the introduction phase, the goal is that all participants should (1) know the recommended weekly exercise target and have reached their individual exercise volume at least 1 week, (2) understand the general benefits of exercise, (3) know how to measure intensity objectively (heart rate) and subjectively (Borg scale, talk test), (4) be able to use their activity watch and App (monitoring, data storage and simple problem solving) and (5) have initiated home-based exercise with subsequent evaluation with study personnel.

During the remaining study period, participants should maintain the exercise volume and habits attained. They have access to a personal eHealth platform provided by MIA Health AS. Each participant also has access to a contact person (coach) that monitor exercise status and progress, give advice and support throughout the intervention. A total of seven coaches have the main responsibility for exercise supervision. In addition to following a written protocol, the coaches have regular meetings to share experience and distribute information. The main communication channel between the patient and coach is through an App and management portal. For self-monitoring of exercise volume participants can opt to use a personalised PA metric called Activity Quotient (AQ, previously PAI) for guidance.^18^ AQ is automatically calculated in the App based on continuous heart rate measurements. By obtaining at least 100 weekly AQ equivalents, the participants are certain to have reached a volume and intensity of exercise aligned to the recommendations. They should also be able to mix and match their individual exercise sessions after personal preferences (type, intensity and frequency) and be aware of facilities and possibilities in their own community. They receive feedback and support through the App, and activity levels are monitored continuously. Patients are also offered monthly group-based exercise sessions. Participants who fall below recommended volume over time or are at risk of dropping out are given closer follow-up through the App or meetings with the aim to tailor goals/programme, motivate and/or identify causes of low adherence.

#### Usual care group

The usual care control group receive standard management according to usual practice at the respective participating centres, including medical therapy as per guidelines. The usual care group received oral information about general PA recommendations as per guidelines at study enrolment. No further supervision is given.

### Outcomes

#### Time in AF

An ICM (Confirm RX, Abbott) is implanted subcutaneously a minimum of 4 weeks prior to baseline testing and randomisation, to continuously monitor the heart rhythm during follow-up. The ICM connects wirelessly to a web-based platform via a smartphone application and provides information on start-time, end-time and duration of all AF episodes as well as heart rate during AF. The ICM is explained at the end of battery life (up to 2 years) or at the participants request. The ability of the ICM to detect true positive AF episodes will be examined and reported in a separate validation study.

#### AF-related QoL

AF-related QoL is measured by the AFEQT questionnaire, a validated 20-item questionnaire designed to assess the impact of AF on patient’s health-related QoL and possible changes with intervention.^19^ Responses are scored on a 1–7 Likert scale, from 1=‘not at all…’ up to 7=‘extremely’. The AFEQT has an overall score (0=complete disability, 100=no disability) and subscale scores in three domains: symptoms, daily activities and treatment concerns. Two questions regarding satisfaction with healthcare providers and with treatment are not included in the overall AFEQT score and are not collected.

#### Other questionnaire-based outcomes

General health-related QoL is measured by the RAND-12 questionnaire, containing 12 items corresponding to 8 principal physical and mental health domains.^20^ The items are summarised into two scores: ‘physical health summary measure (Physical Component Score)’ and ‘mental health summary measure (Mental Component Score). In addition, EQ-5 Visual Analogue Scale is used as an overall health score.^21^

The Atrial Fibrillation Symptoms and Severity Checklist (AFSSC) measures frequency and severity of 16 listed AF symptoms in the last month.^22^ The 16 symptoms listed are tiredness, heart fluttering, heart racing, dizziness, headache, trouble concentrating, hard to catch breath, feeling warm/flushed, sweating, weakness, poor appetite, nausea, difficulty sleeping, chest pain when the heart is fluttering/racing and chest pain when the heart is not fluttering or racing. On the frequency scale, patients report how frequently they have experienced the symptom the last month (never=0, rarely=1, sometimes=2, often=3 or always=4), and on the severity scale, they rate the symptoms as mild=1, moderate=2 or severe=3.

Data on self-reported frequency of PA (‘How often do you exercise?’, with the response options ‘never’, ‘less than once a week’, ‘once a week’, ‘2–3 times a week’ or ‘almost every day’), intensity of PA (How hard do you push yourself?’ with response options ‘I take it easy, I don’t get out of breath or break a sweat’, ‘I push myself until I’m out of breath and break into a sweat’ or ‘I practically exhaust myself’) and duration of PA (‘How long does each session last?’ with response options ‘less than 15 min’, ‘15–29 min’, ‘30 min to 1 hour’ or ‘more than 1 hour’) is collected using a validated questionnaire.^23^ Smoking habits (never, former, sometimes or daily smoker), alcohol use last year (units per month) and sleep patterns (symptoms of sleep apnoea, insomnia and hours of night-time sleep)^24^ are also collected through questionnaire. All questionnaires are distributed digitally at baseline (before randomisation), at 6 months and 12 months. The AFSSC is distributed separately at baseline (1–2 days before randomisation), and together with other questionnaires at 6 months and 12 months. Relevant medical history, medication use, clinical events and procedures will be collected at baseline and after 12 months by interview and review of medical records.

#### Cardiorespiratory fitness

VO_2peak_ is measured by ergospirometry (Vyntus CPX, Carefusion, Germany) and 12-lead ECG (Custo Med) using an individualised incremental treadmill protocol to voluntary exhaustion. Participants undergo an approximately 10 min warm-up at a moderate intensity (gradual increase to ~Borg scale 12), followed by 3 min submaximal steady state measurements at the same workload while wearing a face mask (Hans Rudolph 7450, Hans Rudolph, USA). After the submaximal level, speed (1 km/hour) and/or incline (2%) are increased every minute until exhaustion. Peak heart rate during the test is assessed for personal exercise prescription, and ventilation parameters, subjective rating of perceived exertion (Borg 6–20) and work economy registered in addition to VO_2peak_. The exercise testing is performed in sinus rhythm also at the 12-month follow-up (unless the treatment strategy at follow-up has changed to rate control and/or testing in sinus rhythm is not feasible). All testing personnel were trained at the NextMove Core Facility for exercise science at NTNU and followed a written protocol for CPET measurements.

#### Echocardiography

Indices of cardiac structure and function are assessed by two-dimensional and three-dimensional echocardiography in sinus rhythm by an experienced physician or sonographer. Images and recordings are transferred and stored in a secure database at the core lab at the coordinating centre for analyses by expert personnel (cardiologist or sonographer) blinded to group and time point of the recordings (preintervention or postintervention). Key secondary outcomes are indexed left atrial (LA) end-systolic volume, left-ventricular (LV) end-diastolic volume and LA to LV volume ratio. Measurements of right-ventricular size and function, measures of deformation (strain) and motion of the myocardium, and blood flow measurements will serve as other outcome measures.

#### Cardiovascular risk factors and biomarkers

Body weight (kg), height (cm) and waist circumference (cm) are measured in all participants at baseline and 12 months follow-up. Additional measurements of body composition (muscle mass, body fat and body fat per cent) are measured for a subsample using bioimpedance methodology (InBody 770, BioSpace, Korea). Blood pressure and resting heart rate are measured in a sitting position by an automatic oscillometric method. Systolic and diastolic blood pressures are measured on both arms with 1 min interval. Then a second measurement is taken on the arm with the highest systolic blood pressure. If the difference between the two measurements on the same arm exceeds 10 mm Hg, a third measurement is taken. Serum samples are collected after overnight fasting and 10 min rest at baseline and 12 months. Samples are analysed for high-density lipoprotein, low-density lipoprotein and total cholesterol, triglycerides, glucose, C reactive protein using a high-sensitivity assay (hs-CRP), creatinine and estimated glomerular filtration rate. For a subgroup of participants, two blood samples from baseline and 12 months will be stored in a biobank for future analyses. In addition, serum samples will be taken after maximal exercise testing (0 hour, 3 hours, 24 hours) at baseline and postintervention for later analyses of acute biomarker response to exercise. Preplanned analyses are high-sensitivity-troponins T and I, N-terminal pro-brain natriuretic peptides and inflammatory markers (interleukin-6, hs-CRP). The combined intervention effect on key cardiovascular risk factors will be assessed as a secondary outcome by constructing a continuous cardiometabolic risk score as described by others.^25^

### Statistical analyses

A summary of prespecified primary and secondary endpoints are listed in table 2. The primary analyses of outcomes will be performed using the intention-to-treat principle. The primary analysis of time-in-AF will assess the between-group difference in exercise vs control over the 12-month intervention period using an analysis of covariance model with per cent time-in-AF as the dependent variable and baseline AF burden (4 weeks run-in), study centre and AF subtype (paroxysmal vs persistent) as covariates. As a secondary analysis, we will use a linear mixed model with time-in-AF averaged over each 4-week period as outcome variable and time and postintervention time-by-group interaction as fixed variables. Patient age, study centre and AF subtype will be included as additional covariates. Statistical analysis of the AFEQT will be performed using a linear mixed model with the overall score at baseline, 6 months and 12 months as outcome variable and the intervention effect at 12 months as the primary aim of the analysis. Other secondary endpoints will be analysed analogous to the AFEQT outcome, except incident clinical events and events and procedures that will be reported by intervention group and statistically compared by Poisson regression if appropriate. In case of a data distribution clearly violating statistical assumptions, alternative statistical methodology will be considered. P values <0.05 are selected to indicate statistically significant results.

**Table 2:** Summary of prespecified primary and secondary endpoints of the NEXAF trial

Endpoint	Level	Description	Time frame of endpoint for analysis
Time in AF (%)	Primary	Baseline to 12 months	Baseline to 12 months
Overall AFEQT score	Primary	At baseline, 6 months, 12 months	12 months
Time in AF (%) at month 1–12	Secondary	Continuous from 4 weeks prior to randomisation to 12 months after	Months 1, 2, 3, 12.
No. of AF episodes	Secondary	Continuous from 4 weeks prior to randomisation to 12 months after	Baseline to 12 months
Median episode duration	Secondary	Continuous from 4 weeks prior to randomisation to 12 months after	Baseline to 12 months
Overall AFEQT score	Secondary	At baseline, 6 months and 12 months	6 months
AFEQT subscale: symptoms	Secondary	At baseline, 6 months and 12 months	12 months
AFEQT subscale: daily activities	Secondary	At baseline, 6 months and 12 months	12 months
AFEQT subscale: treatment concern	Secondary	At baseline, 6 months and 12 months	12 months
AF symptoms frequency (AFSSC)	Secondary	At baseline, 6 months and 12 months	12 months
AF symptoms severity (AFSSC)	Secondary	At baseline, 6 months and 12 months	12 months
Peak oxygen uptake (VO_2peak_)	Secondary	At baseline and 12 months	12 months
Physical Component Score (RAND-12)	Secondary	At baseline, 6 months and 12 months	12 months
Mental Component Score (RAND-12)	Secondary	At baseline, 6 months and 12 months	12 months
EQ-VAS	Secondary	At baseline, 6 months and 12 months	12 months
Aggregated cardiovascular risk factors	Secondary	At baseline and 12 months	12 months
Left atrial volume (crude)	Secondary	At baseline and 12 months	12 months
Left atrial volume (BSA indexed)	Secondary	At baseline and 12 months	12 months
Left ventricular volume	Secondary	At baseline and 12 months	12 months
Ratio of left atrial to ventricular volume	Secondary	At baseline and 12 months	12 months
Incident mortality or cardiovascular hospitalisation	Secondary	Baseline to 12 months	12 months
Incident stroke or TIA	Secondary	Baseline to 12 months	12 months
Incident cardioversions	Secondary	Baseline to 12 months	12 months
Incident ablations	Secondary	Baseline to 12 months	12 months
Physical activity level	Secondary	At baseline and 12 months	12 months

AFatrial fibrillationAFEQTAtrial Fibrillation Effect on Quality of LifeAFSSCAtrial Fibrillation Symptoms and Severity ChecklistBSAbody surface areaEQ-VASEuroQoL-Visual Analogue ScaleNEXAFNorwegian Exercise in Atrial Fibrillation TrialTIAtransient ischaemic attack

### Sample size

NEXAF is designed as a superiority trial comparing two parallel groups, exercise in addition to usual care and usual care only (control). The sample size is calculated for the two primary endpoints; the mean time in AF during the 12-month intervention, and the AFEQT sum score at 12 months.

For the mean time-in-AF over 12 months, a 50% relative between-group difference or more at 12 months is considered clinically relevant, translating to a 5 percentage points absolute difference at a baseline AF burden of 10%. We assume the SD to be smaller in the exercise group than the control group during the intervention period due to expected values closer to zero, in line with a recent, similar study with SDs for 4-week averages of around 10 in the exercise group and around 20 in the control group.^13^ Using these SDs, which are probably conservative for a 12-month average, 318 patients (159 in each group) are needed to have at least 80% power (with a significance level of 0.05) to detect a 5 percentage points difference in mean AF-time in exercise versus controls using a two-sample t-test.

For the AFEQT, a difference in sum score of ±5 or more has previously been identified as a clinically important change at the individual patient level.^26^ Previous studies from comparable patient groups have reported SDs of 15–20,^27^ and an SD of 18 is used for sample size calculation. The sample size for AFEQT is estimated by a simulation using 1000 iterations of a linear mixed-model analysis, assuming three data points per participant (baseline, 6 months and 12 months), a treatment effect of 5 points at both follow-up time points, an SD of 18 points and a correlation between repeated measures of 0.5. Based on these assumptions we estimate that 155 patients in each group give a power of 80% (significance level 0.05) to detect a 5-point difference at 12 months follow-up. From these calculations, a total of 320 patients (160 in each group) are considered sufficient.

### Dissemination

At a group level, we believe the general benefits of exercise outweigh potential risks also in patients with AF, although we cannot exclude an unfavourable effect on AF burden in individual patients. Review of medical journals and thorough testing at baseline, including maximal exercise testing with ECG, echocardiography and monitoring of heart rate and rhythm, should minimise the risk of adverse events. A safety monitoring board consisting of two experienced cardiologists and a statistician performed unblinded review of safety data after 100 participants had been included for at least 6 months. The results of the study will be submitted for publication in relevant international peer-reviewed journals and made publicly available through open access or self-archiving.

## Conclusion

The NEXAF trial is designed and powered to provide documentation of long-term exercise effects on total AF burden and disease-specific QoL. Its results may contribute to more specific guidelines on exercise in future AF management strategies.

## Data Availability

No data are available.
